# Topical application of human-derived Ig isotypes for the control of acute respiratory infection evaluated in a human CD89-expressing mouse model

**DOI:** 10.1038/s41385-019-0167-z

**Published:** 2019-05-19

**Authors:** Sandra Koernig, Ian K. Campbell, Charley Mackenzie-Kludas, Alexander Schaub, Marius Loetscher, Wy Ching Ng, Roland Zehnder, Pawel Pelczar, Ildem Sanli, Monther Alhamdoosh, Milica Ng, Lorena E. Brown, Fabian Käsermann, Cédric Vonarburg, Adrian W. Zuercher

**Affiliations:** 1 CSL Limited, Bio21 Institute, 30 Flemington Rd, Parkville, VIC 3010 Australia; 2 Department of Microbiology and Immunology The Peter Doherty Institute for Infection and Immunity, The University of Melbourne, 792 Elizabeth St, Melbourne, VIC 3000 Australia; 3 CSL Behring AG, Wankdorfstrasse 10, 3010 Bern, Switzerland; 4 Center for Transgenic Models, Mattenstrasse 22, 4002 Basel, Switzerland

## Abstract

Recurrent and persistent airway infections remain prevalent in patients with primary immunodeficiency (PID), despite restoration of serum immunoglobulin levels by intravenous or subcutaneous plasma-derived IgG. We investigated the effectiveness of different human Ig isotype preparations to protect mice against influenza when delivered directly to the respiratory mucosa. Four polyvalent Ig preparations from pooled plasma were compared: IgG, monomeric IgA (mIgA), polymeric IgA-containing IgM (IgAM) and IgAM associated with the secretory component (SIgAM). To evaluate these preparations, a transgenic mouse expressing human FcαRI/CD89 within the myeloid lineage was created. CD89 was expressed on all myeloid cells in the lung and blood except eosinophils, reflecting human CD89 expression. Intranasal administration of IgA-containing preparations was less effective than IgG in reducing pulmonary viral titres after infection of mice with A/California/7/09 (Cal7) or the antigenically distant A/Puerto Rico/8/34 (PR8) viruses. However, IgA reduced weight loss and inflammatory mediator expression. Both IgG and IgA protected mice from a lethal dose of PR8 virus and for mIgA, this effect was partially CD89 dependent. Our data support the beneficial effect of topically applied Ig purified from pooled human plasma for controlling circulating and non-circulating influenza virus infections. This may be important for reducing morbidity in PID patients.

## Introduction

Primary immunodeficiency (PID) diseases are a group of heterogeneous diseases with more than 300 genetically defined markers that affect the function of the immune system.^1,2^ In a subset of PID patients, plasma and mucosal immunoglobulin (Ig) levels are reduced or absent, making them more susceptible to infection. To compensate for reduced antibody production, PID patients may receive ongoing inoculations of plasma-derived intravenous Ig (IVIg) or preparations given by the subcutaneous route, often supplemented with prophylactic antibiotics. Commercially available plasma-derived Igs primarily consist of highly purified polyclonal IgG, obtained by fractionation of plasma pooled from thousands of blood donors. Due to their multi-donor origin, the purified IgGs display a broad range of specificities to viral, bacterial and fungal antigens circulating in the general population.

Stable IgG supplementation and antibiotic prophylaxis have successfully reduced persistent infections, such as pneumonia in PID patients,^3^ illustrating the benefit of this approach. Nevertheless, recurrent and persistent infections in both the upper and lower respiratory tracts continue to affect PID patients.^3–5^ The causes of these recurrent infections are not fully understood, but might be a result of local lung immunodeficiency and/or insufficient transudation of the supplemented IgG into the respiratory tract. Indeed, in cynomolgus monkeys, it has been shown that intravenous (i.v.) administration of the monoclonal antibody mepolizumab, a humanised IgG1 monoclonal antibody against IL-5 for the treatment of asthma, results in a 500- to 1000-fold lower concentration of the antibody in the bronchoalveolar lavage fluid (BAL) compared with the steady-state plasma concentration.^6^ Similar findings have been reported in monkeys for humanised anti-respiratory syncytial virus monoclonal antibodies.^7^ In mice, i.v. injection of human IVIg preparations resulted in 60–70% survival after lethal influenza infection, whereas intranasal (i.n.) IgG administration protected 90% of mice at doses 40–100 times lower,^8^ suggesting increased availability and effectiveness of human Ig when applied directly to the mucosal surface.

Assuming that PID patients under appropriate IgG substitution therapy would still have local immunodeficiency in the upper and conductive airways, we hypothesised that protection of these areas of the respiratory tract might be best achieved by direct topical application of Igs to increase the Ig concentration at the site of infection. Although IgG is present in the lung, secretory immunoglobulin A (SIgA) and secretory immunoglobulin M (SIgM) generally provide the primary defence against mucosal pathogens in healthy individuals. Mucosal Igs from healthy individuals cannot be collected in sufficient amounts to evaluate their capacity in protecting the lung from infections after topical application. However, human plasma is collected on a large scale for the purification of numerous plasma-derived products, and plasma IgG, IgA and IgM are present at concentrations of 1050 (range 713–1852), 233 (80–531) and 141 (42–412) mg/dl, respectively,^9^ sufficient for purification and evaluation for topical application.

There are several molecular differences in the Ig species between lung and plasma. In the lung, SIgA comprises dimeric and polymeric forms, of which 90% is of the IgA1 alloform. SIgA and SIgM are complexes comprising the respective Ig, the J-chain and the secretory component (SC). In plasma, IgA consists of 80% monomeric IgA (mIgA) and 20% polymeric IgA (pIgA), including dimeric IgA. The IgA1 and IgA2 alloforms are present at 85% and 15%, respectively.

Two different IgA- and IgM-containing preparations can be purified as part of the fractionation process used to purify IgG for the production of i.v. or subcutaneous Ig, as previously reported.^10,11^ One comprises mIgA and the other pIgA (mostly dimers) together with pentameric IgM (IgAM). A third preparation can be generated by associating SC with IgAM (SIgAM), resembling SIgA and SIgM present at mucosal sites.^10,11^

Polyclonal IgG, obtained by fractionation of pooled plasma, has been shown to contain antibodies that are broadly cross-reactive across different influenza A subtypes.^12–14^ While these cross-reactive antibodies are present at low levels, topical application protected 90% of mice.^8^ Polyclonal IgA-containing preparations have yet to be compared with IgG obtained from the same pool of plasma donors for the presence of cross-reactive anti-influenza antibodies and their capacity to alter the course of infection after topical application.

In humans, IgA binds to numerous receptors, including the FcαRI/CD89, CD71/transferrin receptor, the asioglycoprotein receptor, Fcα/μR, FcRL4, the polymeric immunoglobulin receptor (reviewed in refs. ^15,16^) and possibly the intracellular Trim21.^17^ FcαRI/CD89 is unique amongst these receptors, in that, it is neither expressed in mice nor is there any homologue in mice.^18^ IgA-dependent activation of CD89 is believed to be a key factor in host defence and responses, including phagocytosis, respiratory burst, degranulation and cytokine release.^19–21^ It has also been proposed that although the cross-linking of CD89 during infection with IgA-opsonised pathogens induces pro-inflammatory responses, naturally occurring serum IgA not complexed with an antigen induces inhibitory signals through CD89 to dampen excessive immune responses.^22^ Studies in several CD89 transgenic (Tg) mouse strains have shown that full-length CD89 was functional in mice, with phagocytosis and cytokine release depending on the association with the signalling FcRγ-chain.^18,23^ To date, CD89 Tg mice have not been utilised to evaluate the efficacy of IgA-containing preparations in controlling influenza respiratory tract infections and the severe inflammatory responses that often accompany these infections.

Two H1N1 subtype viruses were chosen for our studies to represent a recently circulating virus and a past isolate, simulating a virus that has not been encountered by the donor population. A/California/7/09 (Cal7) influenza virus has been circulating globally since its emergence as a pandemic isolate in 2009. This strain was recommended by the World Health Organisation to be included in the influenza vaccines between 2010 and 2014 (www.fludb.org). In contrast, A/Puerto Rico/8/34 (PR8) virus was initially isolated in 1934 and is no longer present in the human circulation. PR8 was used here to represent a virus that was unlikely to have been encountered by the donor population.

In this study, we describe a novel CD89 Tg mouse and utilise it to compare the effectiveness of topically applied human polyclonal mIgA, IgAM, SIgAM and IgG preparations for the treatment of currently circulating and antigenically distinct influenza virus infections. We report that IgA-containing preparations can protect mice from lethal influenza infection in both a CD89-dependent and CD89-independent manner, but that IgG provides the greatest protection from disease. We identify influenza strain-specific inflammatory responses in the lungs of influenza-infected mice that were differentially resolved following treatment with the various Ig preparations. Our data suggest that the topical application of IgG and to some extent IgA to the lungs may be beneficial in preventing respiratory tract infections in PID patients.

## Results

### Generation of human CD89-expressing mice

**Fig. 1:**
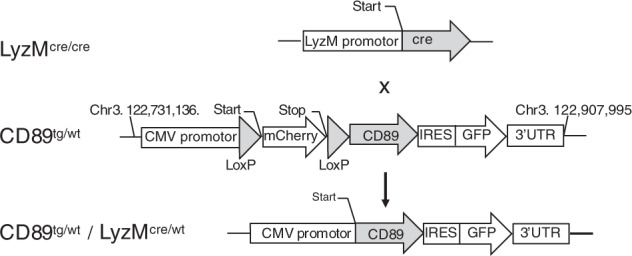
Schematic representation of the cloning strategy used for the generation of CD89 transgenic mice. (Top) Homozygous LyzM^cre/cre^ mice were crossed with heterozygous CD89^tg/wt^ mice (middle), carrying a single cassette containing the CMV promotor, mCherry with a Stop codon flanked by LoxP sites, human CD89, IRES and GFP on chromosome 3. Upon crossing, the CD89^tg/wt^/LyzM^cre/wt^ (bottom) progeny express CD89 under the CMV promotor in the myeloid lineage. Littermates are LyzM^cre/wt^ and negative for CD89 (not shown)

A CD89 Tg mouse line on the C57BL/6 background was generated using a construct containing the full-length human CD89 cDNA followed by an internal ribosomal entry site (IRES) and green fluorescent protein (GFP) cDNA, all downstream of a loxP-flanked mCherry cassette under the control of the CMV promotor (Fig. 1, middle row). Whole-genome sequencing identified a single-copy insertion on chromosome 3, with three nucleotide changes, A161T, G337A and T538C, resulting in two amino acid changes: glutamic acid to valine, E54V, and aspartic acid to asparagine, D114N, in the extracellular domain of CD89 (Suppl. Fig. 1). The insertion event was associated with the genomic deletion of 178 kb on chromosome 3, with no observed breeding effect. To obtain myeloid-specific CD89-expressing mice, heterozygous CD89^tg/wt^ mice were crossed with C57BL/6 LyzM^cre/cre^ Tg mice (Fig. 1, top row) to excise the loxP-flanked mCherry cassette in vivo and drive CD89 expression within the myeloid cell lineage under the CMV promoter in 50% of the offspring (Fig. 1, bottom row). The littermates (LM) CD89^wt/wt^/LyzM^cre/wt^ served as controls for subsequent studies.

**Fig. 2:**
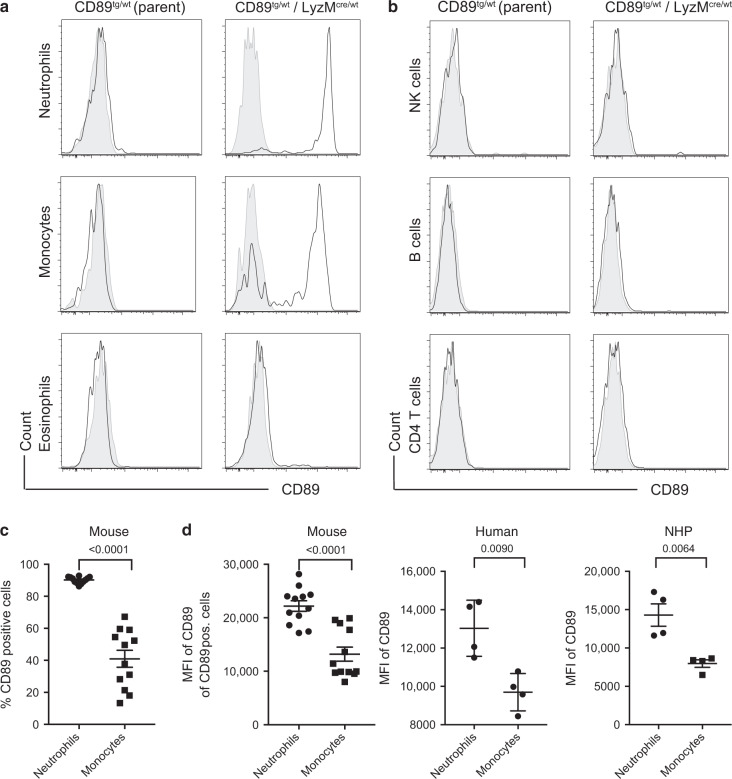
Peripheral blood myeloid cells express CD89. Peripheral blood myeloid cells (**a**) and lymphoid cells (**b**) from indicated genotypic mice were stained with α-CD89, clone A59 (open histogram). Fluorescence-minus-one (FMO) controls are shown (filled histogram). **c** Percentage of CD89-positive cells and **d** CD89 expression levels in CD89^tg/wt^/LyzM^cre/wt^ mice (*n* = 12) and for comparison in human (*n* = 4) and NHP (*n* = 4), peripheral blood neutrophils and monocytes are shown in MFI ± SEM. **c**, **d** *P*-values shown were determined using Student’s paired *t* test

To confirm myeloid-specific CD89 expression in CD89^tg/wt^/LyzM^cre/wt^ mice, flow-cytometric analysis was performed on peripheral blood (PB). CD89 expression was detectable on 90% of neutrophils and on average 40% of monocytes (Fig. 2a, c), but not on lymphoid NK cells, B cells or CD4 T cells (Fig. 2b). CD89 expression was increased on neutrophils compared with monocytes, resembling the human and nonhuman primate system (Fig. 2d). As expected, CD89 expression was associated with reduced mCherry expression, due to excision by the Cre recombinase (Cre) in the myeloid cell lineage (Suppl. Fig. 2a). GFP was undetectable in all cell types (Suppl. Fig. 2).

### Human CD89 is functional in cells of the myeloid lineage of mice

**Fig. 3:**
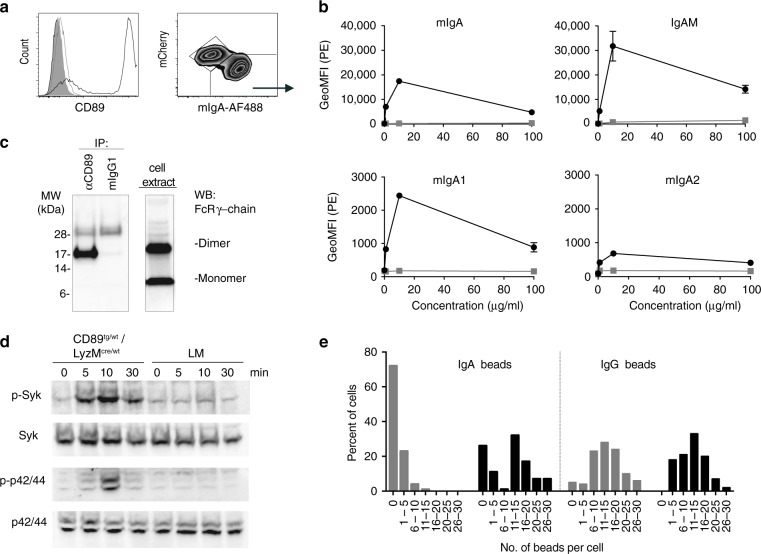
Human CD89 is functional in Tg mice. **a** Flow-cytometric analysis of CD89 expression and mIgA binding on CD89^tg/wt^/LyzM^cre/wt^ (black) and littermate (filled grey) bone marrow-derived macrophages (BMDM). Fluorescence-minus-one (FMO) control is shown in open grey line. Right: Binding of fluorescently labelled polyclonal mIgA is restricted to the mCherry low population. **b** Flow-cytometry binding studies of various human IgA species. BMDM from CD89^tg/wt^/LyzM^cre/wt^ (black) and littermates (grey) were incubated with polyclonal human mIgA and IgAM preparations and monoclonal human IgA1 and IgA2. Bound IgA was detected with αIgA-PE. Geometric MFI ± SD from mCherry low-expressing cells from duplicates are shown. **c** CD89 is associated constitutively with dimeric FcRγ-chain. CD89 was immunoprecipitated (IP) with an anti-CD89 antibody (clone A59) from whole-cell extracts (right panel) prepared from unstimulated BMDM from a CD89^tg/wt^/LyzM^cre/wt^ mouse. Protein was eluted and run on SDS-PAGE under non-reducing conditions. The membrane was probed with an anti-FcRγ chain antibody. **d** CD89 mediates induction of Syk and p42/44 phosphorylation. CD89^tg/wt^/LyzM^cre/wt^ and littermate (LM) BMDM cells were incubated with IgA-coated beads for the indicated times. Samples were run on SDS-PAGE and Syk and p42/44 phosphorylation were determined using phospho-specific antibodies. The same membrane was reprobed with anti-Syk and anti-p42/44 antibodies. **e** CD89 facilitates uptake of IgA-coated beads. CD89^tg/wt^/LyzM^cre/wt^ (black) and littermate (grey) BMDMs cultured in eight-well slides were incubated at 37 °C with IgA- or IgG-coated beads at a cell-to-bead ratio of 25:1 for 30 min. Slides were stained with HOECHST and anti-kappa-PE to visualise nuclei and external beads and imaged. The number of internalised beads per cell were counted manually. Over 100 BMDM cells per stimulus were counted. The percentage of cells with internalised numbers of beads are shown

CD89 functionality in CD89^tg/wt^/LyzM^cre/wt^ mice was determined using bone marrow-derived macrophages (BMDM) as a cellular source. CD89 expression and the binding of human IgA classes were evaluated by flow cytometry using fluorescently labelled IgA or an anti-human IgA antibody. CD89^tg/wt^/LyzM^cre/wt^ BMDM expressed high levels of CD89 (Fig. 3a, left panel) and bound polyclonal human mIgA in the mCherry^low^ population (Fig. 3a, right panel). Human CD89 bound all classes of human IgA: mIgA and pIgA in a mixed IgA/IgM preparation (IgAM, as described in Suppl. Table I), and both mIgA isotypes, IgA1 and IgA2 (Fig. 3b). At equal protein concentrations, CD89 preferentially bound the pIgA species present in the polyclonal IgAM preparation (65% IgA) compared with the mIgA species (98% IgA), as indicated by the higher mean fluorescence intensity (MFI). The IgAM preparation contained higher amounts of multimeric Igs (Suppl. Table I), suggesting that an avidity effect may be contributing to the enhanced binding. Furthermore, IgA1 was preferentially bound compared with IgA2 (Fig. 3b).

Next, CD89-mediated signalling was evaluated by determining the association with the signalling facilitating the FcRγ-chain, phosphorylation of downstream molecules and confirming IgA-mediated phagocytosis. Membrane extracts from steady-state BMDM were immunoprecipitated with anti-CD89 mAb (clone A59) or control mIgG, enriched with Protein G and compared with total membrane extracts following SDS-PAGE under non-reducing conditions. Western blotting using an anti-FcRγ-chain Ab confirmed binding of the FcRγ-chain to CD89, with the dimeric form preferentially bound to the receptor as expected (Fig. 3c). Exposure of BMDM from CD89^tg/wt^/LyzM^cre/wt^ and LM mice to IgA-coated beads induced phosphorylation in Syk and Erk in a CD89- and time-dependent manner (Fig. 3d).

To examine receptor-mediated phagocytosis, BMDM from CD89^tg/wt^/LyzM^cre/wt^ and LM mice were incubated with mIgA-coated beads for 30 min, and internalised beads were enumerated by microscopy. IgG-coated beads served as the positive control for FcγR-mediated uptake. Similar numbers of IgG-coated beads were taken up per cell by both types of BMDMs, with a central peak at 11–15 beads per cell (Fig. 3e). In comparison, 72% of BMDM lacking CD89 contained no internalised IgA-coated beads and only 1% of BMDM contained more than 11 beads per cell. In contrast, the IgA bead uptake in CD89^tg/wt^/LyzM^cre/wt^ BMDM was biphasic. Sixty-three percent of BMDM contained more than 11 beads per cell, suggesting CD89-mediated uptake. Twenty-six percent of CD89^tg/wt^/LyzM^cre/wt^ BMDM failed to phagocytose IgA-coated beads, likely representing the mCherry^high^ expressing cells that do not express CD89 (Fig. 3a).

### Cross-linking of human CD89 results in cytokine production

**Fig. 4:**
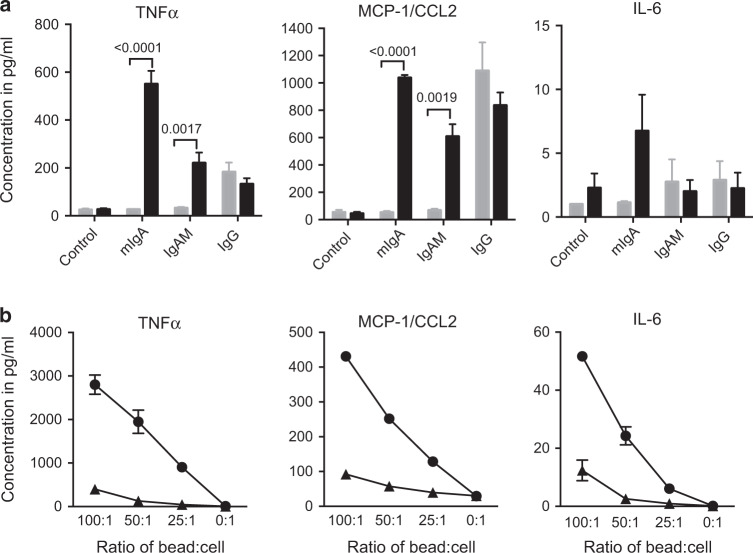
Human CD89 mediates cytokine production by BMDM. **a** BMDM from CD89^tg/wt^/LyzM^cre/wt^ (black) and littermates (grey) were cultured for 24 h on surface-bound polyclonal mIgA, IgAM or IgG. Cytokine concentrations in pg/ml (mean ± SEM, *n* = 3 individual mice) are shown. The effect of the CD89 protein was tested using a two-way ANOVA with Sidak’s multiple comparison test. **b** CD89^tg/wt^/LyzM^cre/wt^ BMDM were incubated overnight with mIgA-coated (●) or BSA-coated beads (▲) at varying ratios. Cytokine concentrations in pg/ml (means ± SD) from duplicates are shown

To further evaluate CD89 functionality, the ability of CD89 to induce cytokine production, when cross-linked with IgA, was examined in vitro using immobilised Ig either on the surface of 96-well plates (Fig. 4a) or on beads in suspension (Fig. 4b). TNFα, CCL2/MCP-1 and to a lesser degree IL-6 were induced upon exposure to surface-bound IgA, when BMDM expressed CD89 (Fig. 4a, b). CD89^tg/wt^/LyzM^cre/wt^ BMDM produced quantitatively more cytokines in response to the polyclonal mIgA preparation (98% IgA), compared with the polyclonal IgAM preparation (65% IgA), likely due to the higher IgA content (Suppl. Table I). As a positive control, we applied surface-bound IgG. As expected, IgG induced equal quantities of TNFα and CCL2/MCP-1 in both types of BMDM, independently of CD89 expression (Fig. 4a).

### Lung myeloid cells express CD89 and bind IgA

**Fig. 5:**
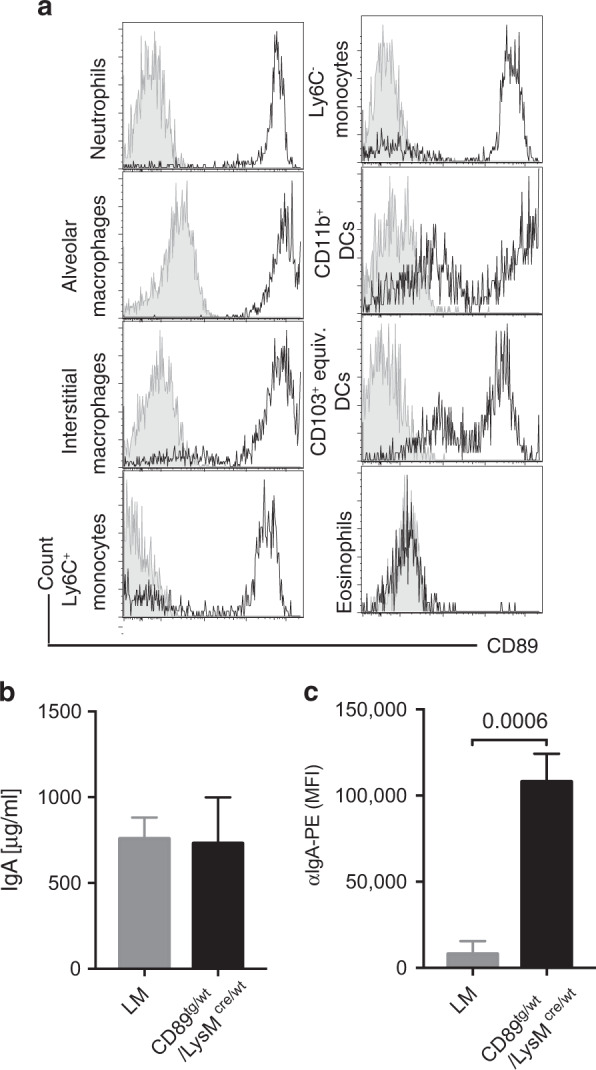
CD89 expression in lung and intranasal delivery of mIgA. **a** CD89^tg/wt^/LyzM^cre/wt^ lung cells were stained with α-CD89 antibody, clone A59 (black) and gated as per Suppl. Fig. 3. Fluorescence-minus-one (FMO) controls are shown in grey. **b** In total, 2.5 mg of mIgA preparation was administered intranasally to CD89^tg/wt^/LyzM^cre/wt^ (black) and littermate control (grey) mice. After 24 h, the BAL was recovered. Data show the human mIgA concentration measured by ELISA in BAL of *n* = 3 mice. **c** Human IgA bound to alveolar macrophages was detected by flow cytometry. Error bars indicate SEM

In order to better understand the role that CD89/IgA interactions might play in mouse lung infection models, we next examined in detail the CD89 expression in lung myeloid cells of CD89^tg/wt^/LyzM^cre/wt^ mice and the effect of CD89 on i.n.-administered IgA. We observed strong CD89 expression on neutrophils, alveolar and interstitial macrophages, monocytes and dendritic cells, but not eosinophils (Fig. 5a, gating strategy in Suppl. Fig. 3). Intranasal delivery of mIgA (2.5 mg) into the lung occurred independently of CD89 expression (Fig. 5b), but was accompanied with preferential binding to CD89-expressing alveolar macrophages (AMφ) (Fig. 5c).

### Ig preparations neutralise influenza activity in vitro

**Fig. 6:**
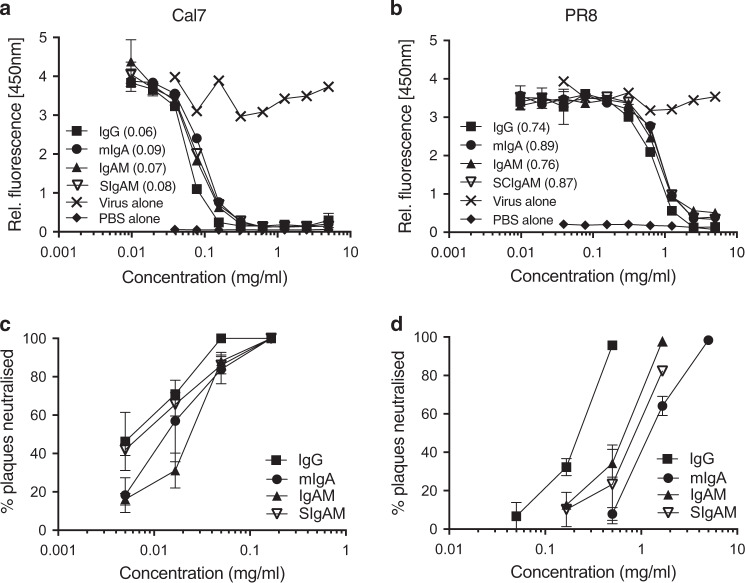
Polyclonal human Ig preparations inhibit Cal7 (**a**, **c**) and PR8 (**b**, **d**) H1N1 influenza neuraminidase activity and virus plaque formation in vitro. **a**, **b** Neuraminidase activity assay. IC_50_ concentrations are shown in brackets. **c**, **d** MDCK virus plaque neutralisation assay. Error bars indicate SD

To determine whether polyclonal Ig preparations from healthy plasma donors could be applied to prevent currently circulating and antigenically distant influenza infections in vivo, we first tested all four Ig preparations against Cal7 and PR8 viruses in vitro for the presence of anti-hemagglutinin antibodies in a hemagglutinin inhibition (HI) assay, for neuraminidase (NA)-inhibiting antibodies in a fluorescence-based assay and for virus-neutralising antibodies in a plaque reduction assay. All four polyclonal Ig preparations (adjusted to 50 mg/ml) inhibited Cal7 with HI titres of 320 (IgG, mIgA and IgAM) or 160 (SIgAM) (Table 1). As expected, HI titres against PR8 were lower than those against Cal7, but still surprisingly substantial for the IgG and mIgA preparations, which showed titres of 80 and 40, respectively (Table 1). All four polyclonal Ig preparations could inhibit the sialidase function of Cal7 and PR8 NA, with only small quantitative differences between the different Igs for a given virus, as reflected in the IC_50_ values (Fig. 6a, b; IC_50_ in brackets). However, the neuraminidase-inhibiting antibody titres of the preparations were tenfold higher against Cal7 than PR8 virus (Fig. 6a, b; the IC_50_ range between 0.06–0.09 mg/ml and 0.74–0.89, respectively). Similarly, all Ig preparations had a greater capacity to neutralise Cal7 virus than PR8 virus (Fig. 6c, d). Cal7 plaques were completely neutralised at 0.2 mg/ml with all Ig preparations (Fig. 6c), while higher concentrations of all preparations were required to neutralise PR8 virus (Fig. 6d). The IgG preparation was significantly more effective in neutralising PR8 virus (~3 to 6-fold greater) than the IgA-containing preparations (Fig. 6d), in line with its higher HI titre.

**Table 1:** Reactivity of human polyclonal Igs to Cal7 and PR8 H1N1 strain

Ig^a^	Cal7^b^	PR8^b^
IgG	320	80
mIgA	320	40
IgAM	320	Nil
SIgAM	160	Nil

^a^Ig preparations (50 mg/ml) from plasma collected between 2011 and 2013 were tested in a standard hemagglutination inhibition (HI) assay

^b^Titres represent the reciprocal of the highest dilution inhibiting four hemagglutinating units of chicken red blood cells

### Human polyclonal IgA protects from influenza-induced weight loss in a CD89-dependent manner

**Fig. 7:**
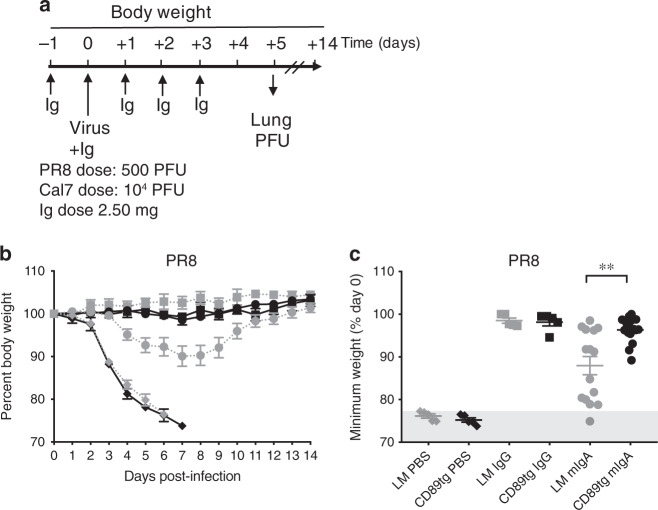
Human polyclonal mIgA protects mice from PR8 H1N1-induced death in a CD89-dependent manner. In total, 2.5 mg of Ig preparation was administered i.n. as indicated in (**a**) and mice were infected i.n. with 500 PFU of PR8 H1N1 on day 0. **b** Percentage of body weight of CD89^tg/wt^/LyzM^cre/wt^ (black) and littermates (grey) treated with IgG (square), mIgA (circle) or PBS (diamond). **c** Minimum weight as a percentage of day 0. Mean ± SEM from two pooled experiments (*n* = 5–15 mice). The shaded area indicates mice euthanised at the humane endpoint. Unpaired *t* test was performed to compare CD89^tg/wt^/LyzM^cre/wt^ and littermates

In order to understand the effect of polyclonal Ig formulations, specifically the prototypic mIgA and IgG formulations, in controlling respiratory tract infection and to determine the role of CD89 in this, we infected CD89^tg/wt^/LyzM^cre/wt^ and LM mice with a lethal dose of PR8 virus, and survival and body weight were observed over the course of 14 days. Groups of mice were treated with either mIgA or IgG preparations according to the schedule in Fig. 7a. All untreated mice reached the predetermined humane endpoint and were euthanised by day 7 (Fig. 7b). mIgA provided only partial protection in LM mice, with 1/15 mice reaching the humane endpoint (Fig. 7). In contrast, mIgA completely protected CD89^tg/wt^/LyzM^cre/wt^ mice from PR8-induced weight loss and death. Indeed, human CD89 expression provided a significant benefit in protection from weight loss compared with LM mice (Fig. 7c). As expected, CD89 expression had no effect on IgG treatment, which completely protected LM and CD89^tg/wt^/LyzM^cre/wt^ mice from PR8-induced weight loss and death (Fig. 7). These data clearly demonstrate the critical role of CD89 in IgA-mediated protection from influenza infection. Accordingly, only CD89^tg/wt^/LyzM^cre/wt^ were used in subsequent studies, as these mice enabled the most equitable comparison between IgA and IgG-containing Ig preparations.

### Human polyclonal Igs protect from influenza-induced weight loss and reduce lung viral titres in an Ig class-dependent manner

**Fig. 8:**
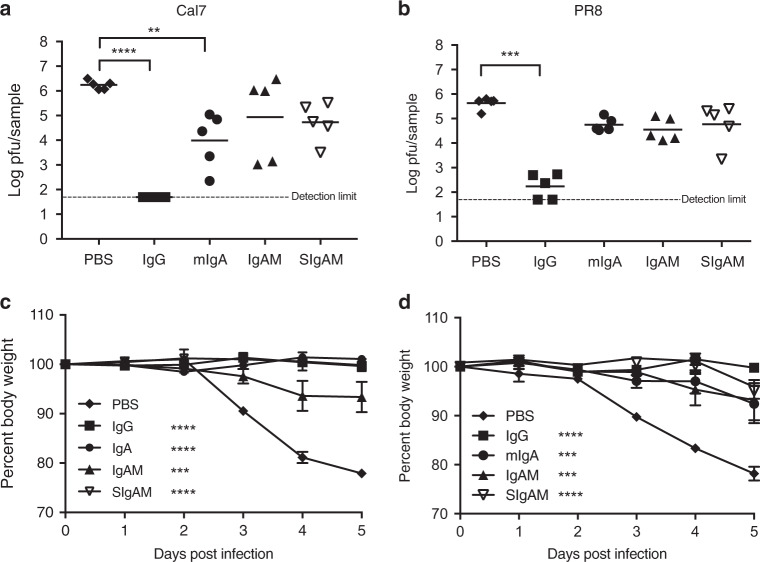
Human polyclonal Igs protect from weight loss and IgG reduces Cal7 and PR8 H1N1 viral titres in vivo. In total, 2.5 mg of Ig preparation was administered i.n. to CD89^tg/wt^/LyzM^cre/wt^ mice and mice infected i.n. with H1N1 influenza on day 0 as indicated in Fig. 7a. Lung viral titres at day 5 (**a**, **b**) and weight (**c**, **d**) after i.n. inoculation of 10^4^ PFU Cal7 (**a**, **c**) and 500 PFU PR8 H1N1 (**b**, **d**) on day 0 are shown. Mean ± SEM values from one experiment are shown (*n* = 5) for each virus. One-way ANOVA with Dunn’s multiple comparison to the PBS group is shown. **P < 0.01, ***P < 0.001, ****P < 0.0001

Next, all four different Ig formulations were examined for their ability to control both Cal7 and PR8 infections in CD89^tg/wt^/LyzM^cre/wt^ mice only. Influenza virus was administered to mice by i.n. application of 10^4^ PFU Cal7 or 500 PFU PR8 (lethal doses), 1 day after initiation of the Ig administration regime (Fig. 7a). PBS-treated mice served as an untreated control. The progression of disease was clinically assessed through weight measurements up to day 5, when the lungs were sampled and homogenates prepared for analysis of viral titres, mRNA transcriptional profile and protein levels of pro-inflammatory cytokines. Cal7 virus was undetectable in the lungs of mice treated with the IgG preparation, whereas mice treated with the IgA-containing preparations still had virus in the lungs on day 5 (Fig. 8a). On average, these levels were at least a log lower than the control group, but only the mIgA preparation showed a statistically significant reduction, due to the spread of titres within the groups (Fig. 8a). A significant reduction in pulmonary viral load after PR8 infection was clearly observed in all IgG-treated mice, while only some mice appeared to benefit from the treatment with the IgA-containing preparations (Fig. 8b). Mice treated with the IgA-containing preparations, while not significantly reducing viral load, were less severely affected by the infection, as shown in the significantly reduced weight loss during Cal7 and PR8 virus infection (Fig. 8c, d).

### Human polyclonal Igs reduce influenza-induced cytokine/chemokine expression in the lung

**Fig. 9:**
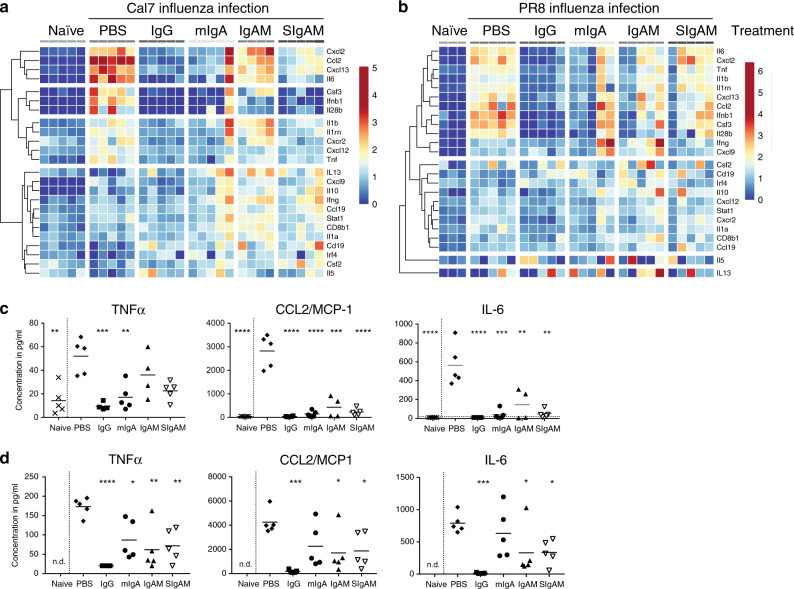
Human polyclonal Igs reduce influenza-induced transcriptional profile and protein levels in the lung. mRNA transcriptional levels (**a**, **b**) and protein cytokine levels (**c**, **d**) of homogenised lungs at day 5 after i.n. inoculation of 10^4^ PFU Cal7 (**a**, **c**) and 500 PFU PR8 (**b**, **d**) H1N1 and Ig treatment as indicated in Fig. 7a are shown. CNRQ, calibrated normalised relative quantities from *n* = 3–5 animals are shown for each experiment. Mean protein concentrations from one experiment are shown (*n* = 4–5) for each virus. One-way ANOVA with Dunnet’s multiple comparison to the PBS group is shown. **P < 0.01, ***P < 0.001, ****P < 0.0001, n.d. not done

Lower levels of pro-inflammatory cytokines/chemokines were observed at both the transcriptional and protein levels in lung extracts of IgG- and mIgA-treated Cal7-infected mice (Fig. 9a, c). In detail, 24 genes were analysed, including cytokines and chemokines (18 genes), cell-signalling components (*stat1*, *irf4*), cytokine receptors (*cxcr2*, *cxcr4*) and cell-type-specific genes (CD8 T cells, *cd8b1*; B cells, *cd19*; neutrophils, *elane*) and normalised to two housekeeping genes (*tbp*, *ubc*). Twelve pro-inflammatory cytokine/chemokine genes, including those encoding TNFα, CCL2, IL-6 and the anti-inflammatory IL-1RA (*il1rn*), were upregulated in Cal7-infected lungs compared with naive lungs and downregulated in 5/5 IgG- and 4/5 IgA-treated mice (Fig. 9a, top three branches). With IgAM and SIgAM, similar trends of reduced Cal7-induced transcriptional levels were observed. In a separate cluster, genes encoding IL-13, IFNγ, CXCL9 and CD8b were increased in mIgA, IgAM and SIgAM-treated animals infected with Cal7 virus (Fig. 9a, bottom branch). Neutrophil-specific genes (*elane*, *cxcr4*) were not detected in naive or infected mice, likely due to the low intrinsic transcriptional activity of this cell type. The lower transcriptional levels of pro-inflammatory cytokines were also evident in the marked reduction of TNFα, CCL2/MCP-1 and IL-6 protein levels for all Ig-treated groups (Fig. 9c).

A similar picture emerged for the transcriptional profile of PR8-infected and Ig-treated mice (Fig. 9b). Twelve genes that were upregulated in PR8-infected lungs compared with naive lungs (Fig. 9b, top branch, excluding *cxcl9* and *ifng*) were downregulated in 5/5 IgG- and 3/5 mIgA-treated mice. An additional large number of genes was unchanged with PR8 infection or Ig treatment (Fig. 8b, lower branches). Similarly, CCL2/MCP-1 and IL-6 protein levels were significantly reduced in PR8-infected mice treated with IgG, IgAM or SIgAM (Fig. 8d). In addition, we observed a statistically significant reduction in the TNFα protein for all Ig-treated, PR8-infected groups.

## Discussion

PID patients continue to experience recurrent and persistent infections in both the upper and lower respiratory tracts, despite stable IVIg supplementation.^3–5^ Drug delivery directly into the respiratory tract to prevent or treat pulmonary diseases offers many benefits,^24^ primarily the localised delivery of high drug concentrations to the affected regions. Ramisse et al.^8^ showed previously that i.n. administration of polyvalent IgG, collected from pooled human plasma, was superior to i.v. administration at significantly lower doses in protecting mice against lethal H3N2 infection. Polyclonal IgG is already broadly utilised in PID and neurological indications. mIgA and IgAM are side products of the IgG fractionation process with relatively little exploited potential to date. The interest in these Igs has grown recently, due to the potential availability of large quantities and natural occurrence of SIgAM at mucosal surfaces.^25^

In this study, we tested one mIgA- and two IgAM-containing preparations and benchmarked them against IgG, which has been extensively tested in influenza infections.^8^ We developed a novel CD89 Tg mouse to allow the unbiased comparison of the four Ig preparations in controlling Cal7 and PR8 influenza infections in vivo. Several Tg mice have been described, in which human CD89 expression was driven either by its own regulatory sequences,^18^ the CD11b promoter^26^ or the CD14 promoter.^27^ In our CD89 Tg mice, CD89 was expressed in myeloid lineage cells under the control of the CMV promoter. We found that the CD89/FcRγ-chain complex mediated downstream phosphorylation of Syk and Erk and induction of pro-inflammatory cytokines upon cross-linking with surface-bound IgA, in line with previous reports.^23,28^ In our mice, the IRES element was inefficient at facilitating GFP expression in accordance with previous findings,^29^ but this was not further investigated as the CD89 expression was, as expected. Our CD89 Tg mouse does not express the CD89 receptor on eosinophils, which resembles the absent or very low receptor expression in human PB eosinophils (Suppl. Fig. 4). With these similarities to human expression and function of CD89, the mouse model described here represents a valuable novel tool to study IgA–CD89 interactions. To our knowledge, we are the first to characterise the CD89 expression pattern in the lungs of a CD89 Tg mouse and also to investigate the role of IgA-containing preparations in a mucosal influenza infection model in the presence of CD89.

All four Ig preparations from plasma collected over the same time period contained higher antibody titres to the recently circulating Cal7 virus compared with the 1934 PR8 virus, as demonstrated by HI assay (Table 1), MDCK virus neutralisation and NA inhibition assays (Fig. 6). While the HI and NA assays did not differentiate between the Ig formulations, IgG showed significantly greater reduction of PR8 viral replication in the plaque neutralisation assay compared with the mIgA- and IgAM-containing formulations. These Ig preparations were therefore suitable candidates to be evaluated for the prevention of influenza infection in vivo. In our CD89 Tg model, topically applied IgG was the most effective preparation in reducing Cal7 and PR8 lung viral titres (Fig. 8a, b) and influenza-induced cytokines (Fig. 9). Nevertheless, the IgA-containing formulation also protected Cal7- and PR8-infected mice from weight loss (Fig. 8c, d) and significantly reduced influenza-induced cytokine and chemokine responses (Fig. 9). The latter orchestrate the fine balance between immune-mediated protection and lung tissue damage. For example, the transcriptional type I/III interferon genes (i.e. Ifnb1 and Il28b), which were greatly upregulated in infected untreated mice, in agreement with a previous publication,^30^ were markedly reduced in all IgG-treated mice and reduced to baseline in 60–80% mIgA-treated mice infected with Cal7 and PR8.

Furthermore, topically applied mIgA protected mice from PR8-induced death (Fig. 7), despite the low HI titre. Ramisse et al.^8^ showed that i.n. administration of IgG or F(ab′)_2_ was similarly protective, indicating that neutralisation was primarily due to a non-Fc-mediated mechanism. However, in our evaluation of mIgA in the PR8 infection model, protection was partially CD89 dependent, as shown by further reduced weight loss in CD89 Tg mice compared with the LM controls (Fig. 7). This suggests that for mIgA at least, both Fab-mediated neutralisation and Fc-mediated effector functions facilitate virus control and reduce disease severity, potentially through modulation of the inflammatory response. Indeed, CD89 has been reported to have a dual role by also providing anti-inflammatory signalling upon monovalent binding of IgA.^28,31^ This anti-inflammatory feature of CD89 was not investigated directly in this study, but it might in part facilitate the significant reduction in cytokines observed with the IgA-containing preparations.

mIgA is normally not present in the lumen of the mucosa. However, it will be functionally relevant because of its direct neutralising capacity. Importantly, IgA can facilitate antibody-dependent cellular phagocytosis and antibody-dependent cellular cytotoxicity.^32^ Due to its size, mIgA should have greater diffusion than pIgA, sIgA and IgM. Together with the FcαRI/CD89-mediated anti-inflammatory effects,^28^ mIgA could protect host tissues from an excessive injury.

In our CD89 Tg mice, CD89 was expressed on AMφ and bound mIgA in a CD89-dependent manner (Fig. 5). He et al.^33^ recently reported that AMφ were critical for broadly reactive antibody-mediated protection against influenza A virus in WT mice. We re-evaluated published microarray data from human AMφ^34^ for the presence of IgA receptors and identified CD89 transcripts, albeit at low levels, in healthy humans (data not shown). WT mice therefore do not fully reflect the IgA receptor repertoire of human AMφ, while our CD89 Tg mice capture this additional aspect of the IgA biology present in humans.

Across the IgA-containing preparations, in vivo, the mIgA preparation reduced viral titres and cytokine levels more effectively than the IgAM-containing preparations, despite similar neutralisation capacities in vitro. The molecular mechanism was not further investigated in this study, but could in part be due to the lower IgA content in the IgAM preparation compared with that in the mIgA preparation (Suppl. Table I). In our hands, addition of the SC to the IgAM formulation provided neither further benefit in virus neutralisation in vitro nor in reducing viral titres in vivo, but marginally reduced cytokine responses in Cal7-infected lungs in vivo (Fig. 9a). It was previously reported that the addition of SC to pIgA confers increased stability on the resulting SIgA,^35,36^ apparently by masking proteolytic sites from proteases present in mucosal secretions. However, the lack of functional benefit of the SC in neutralising influenza that we observed was consistent with a report by Renegar.^36^

Ramisse et al.^8,13,37^ have shown that human polyclonal IgG delivery into the lungs in rodent models can protect from influenza infection. In our studies, we delivered a total of 12.5 mg to the mice, which is in the range of a single dose for a PID patient, receiving 200–800 mg/kg of IVIg. However, relatively little is known about the feasibility of Ig delivery into the lungs of non-human primates (NHP) and humans. Vonarburg et al. (manuscript in preparation) tested the nebulisation of the IgG, mIgA and IgAM formulations described in this study using an optimised Pari nebuliser in rats and NHPs. They found that the IgG and mIgA formulations, and the higher-molecular-weight IgAM formulation, were equally well nebulised when tested at 50 mg/ml. After inhalation, the recovery of nebulised Ig peaked immediately post delivery in the lungs of NHP, suggesting that the inhalation of at least three out of the four Ig preparations tested in our study, can be topically delivered to humans.

In summary, we investigated the effectiveness of different human Ig isotype preparations, prepared from plasma collected over the same time period, to protect mice against influenza when delivered directly to the respiratory mucosa. To our knowledge, we are the first to use CD89 Tg mice in this context, to enable a more equitable comparison between IgA- and IgG-containing preparations. We showed that topically applied mIgA was beneficial in controlling influenza virus infection and that this occurred in a partially CD89-dependent manner; however, IgG provided the greatest protection from disease. Our data support the concept of topical IgG delivery for the treatment of respiratory tract infections in susceptible individuals, such as PID patients.

## Materials and methods

### Generation of a FcaRI/CD89 Tg mouse

Tissue-specific inducible CD89 Tg mice were generated by Dr. Pawel Pelczar (University of Zurich, Switzerland) under a contract. The *CD89* gene (Gene Reference: X54150.1) was inserted into a XhoI restriction site downstream of a floxed mCherry with stop codon (pCAG-loxP-*mcherry*-stop-loxP-XhoI site-IRES-*egfp*) vector. Five mCherry-positive F0 founders, named CD89 Tg, were generated by pronuclear injection of C57Bl/6 J oocytes. Two of the  transgenic founder lines were furthered to establish heterozygous mouse lines (labelled CD89^tg/wt^). Both lines were found to contain identical transgene sequences by Sanger sequencing and expressed similar levels of hCD89 when crossed to the LyzM–Cre driver line.

Myeloid-specific CD89-expressing mice were generated by crossing CD89^tg/wt^ and C57BL/6 LyzM^cre/cre^ Tg mice (licenced from Jackson Laboratories) to excise the loxP-flanked mCherry cassette in vivo and drive CD89 expression in the myeloid cell lineage under a CMV promoter in 50% of the offspring (CD89^tg/wt^/LyzM^cre/wt^). LM (CD89^wt/wt^/LyzM^cre/wt^) served as wild-type (WT) controls. The CD89 gene-containing offspring were detected by PCR using transgene-specific primers (5′-CCAAACAGACACCCTCCTGT-3′ and 5′-GAGGCTTCCTTGTTCAGTGC-3′). All mice were bred at the University of Melbourne Bio21 Institute animal facility under specific pathogen-free conditions.

### Antibody treatment of mice

Eight to ten-week-old mice were anaesthetised with ketamine/xylazine (100 mg/kg and 20 mg/kg, respectively) solution, and 2.5 mg of mIgA (50 μl of a 5% solution) or 50 μl of PBS was applied i.n. After 24 h, mice were euthanised with 400 mg/kg pentobarbital i.p. BAL fluids were obtained by cannulation of the trachea and administration of 3 × 0.4 ml of PBS (0.2 U of heparin) washes. The BAL was separated from the cellular compartment by a 5-min centrifugation at 5000 rpm at 4 °C. The erythrocytes in the cell pellet were lysed and the remaining cells were analysed by flow cytometry (LSR Fortessa, Beckon Dickinson). Bound IgA was detected with PE-conjugated F(ab)_2_-anti-human IgA. Macrophages were gated as F4/80^+^/CD11b^+^/Ly6G^–^ and expressed high levels of CD64, CD89 and CD16/32. IgA in BAL was detected by ELISA (Supplementary Methods).

### Influenza infection and antibody treatment of mice

Influenza infections were performed on 8–10-week-old mice at the Department of Microbiology and Immunology, University of Melbourne. To establish a total respiratory tract infection, mice were lightly anaesthetised by isoflurane inhalation, and 50 μl containing 10^4^ plaque-forming units (PFU) Cal7 or 500 PFU PR8 (both lethal doses) were delivered to the external nares to be breathed in deeply by the animal. To deliver the Ig preparations, 2.5 mg of Ig preparation (50μl of 5% solution) were administered under similar conditions on day –1, day 0 (8 h before viral challenge), day 1, day 2 and day 3. On day 5 post infection, the lungs were sampled, and supernatants from homogenates were prepared. In separate experiments, mice were monitored for a period of 14 days after infection. Mice were euthanised if a predetermined humane endpoint, based on the degree of weight loss and clinical signs, was reached.

### Immunoprecipitation, SDS-PAGE and immunoblotting

Immunoprecipitation and western blotting were performed, as previously described.^38^ In short, membrane extracts were isolated from steady-state BMDM with RIPA solution containing protease inhibitors (cOmplete inhibitors, Roche) at 10^6^ cells per 100 µl for 1 h at 4 °C. Cell nuclei were removed by centrifugation at 1700 rpm for 5 min. Protein content in the supernatant was assessed with the bicinchoninic acid assay (Thermofisher). In multiple experiments, equal amounts of proteins (500–1000 μg) were incubated with either 2 μg of mAb anti-CD89 (clone A59) or control mouse IgG1 for 1 h. Ab complexes were incubated with Protein G-conjugated Sepharose beads (GE) overnight. After three washes with Tris-buffered saline (TBS) solution containing 0.05% Tween 20, the beads were treated with non-reducing SDS buffer and the eluted proteins were separated under non-denaturing conditions on a 4–12% Bis-Tris acrylamide gel. The total lysate was run as positive controls. Proteins were transferred onto a nitrocellulose membrane. After blocking with 2% BSA/TBS/Tween, the FcRγ-chain was detected with polyclonal rabbit anti-FcRγ-chain (Merck Millipore) and horseradish peroxidase-conjugated goat anti-rabbit Ab (DAKO). Proteins were detected by enhanced chemoluminescence (ECL, Amersham-Pharmacia Biotech) using a Chemodoc (Biorad).

### Cytokine production and phosphorylation after Fc receptor cross-linking

Flat-bottom 96-well tissue culture plates were coated overnight with 50 μl of 0.2 M carbonate buffer, pH 9.5, containing 20 μg/ml mIgA, IgAM or IVIg. The next day, plates were washed twice with PBS and once with c-RPMI and seeded with 100,000 BMDM in 200 μl of c-RPMI in triplicate. Alternatively, 100,000 BMDM were incubated with IgA- or BSA-coated Polybeads (2.8 μm, Polysciences) at different ratios. Cells were cultured at 37 °C and 5% CO_2_ for 24 h. Cytokine/chemokine concentrations in supernatants were determined by cytometric bead array (Becton Dickinson). In a separate experiment, aliquots of 2 × 10^6^ BMDM were incubated with 50 × 10^6^ mIgA-coated Polybeads in a 37 °C water bath. At various intervals, the reactions were stopped with 10 ml of ice-cold PBS, followed by an additional wash. The cell pellet was lysed with 4× non-reducing SDS buffer and stored at −80 °C until further analysis. SDS-PAGE and protein transfer were performed as described above. Phosphorylated Syk was detected with polyclonal rabbit anti-phospo-Syk Ab (sc-293118, Santa Cruz) and with HRP-conjugated goat anti-rabbit Ab (P0448, DAKO). After each detection step, primary and secondary Abs were removed using Restore™ western blot stripping buffer (ThermoFisher). The total Syk was detected with polyclonal rabbit anti-Syk Ab (sc-1077, Santa Cruz) and HRP-conjugated goat anti-rabbit Ab (DAKO). Thereafter, phosphorylated and total MAPK/Erk were detected on the same membrane using mouse anti-phospho-p-44/42 MAPK/Erk Ab (Cat. No. 4370, Cell signalling) followed by HRP-sheep anti-mouse Ab, then polyclonal rabbit anti-MAPK/Erk Ab (Cat. No. 9107, Cell signalling) followed by HRP goat anti-rabbit Ab. Proteins were detected by ECL (Amersham-Pharmacia Biotech) using a Chemodoc (Biorad).

### Flow-cytometry staining in mice, human and nonhuman primate cells

The expression of CD89 was analysed by flow cytometry (LSR Fortessa, Beckon Dickenson) using the clone A59. In mice, PB cells were gated as neutrophils (Ly6G^+^, CD11b^+^), monocytes (Ly6C^+^, CD11b^+^), eosinophils (F4/80^+^, CD11b^+^ and SSC^hi^), NK cells (NK1.1^+^, CD3^–^) and CD4 T cells (CD3^+^, CD4^+^). To evaluate lung tissue, mice were perfused. The lung tissue was disrupted with an RPMI solution containing 3.5 mg/ml collagenase D (Sigma) and 0.1 mg/ml DNase I (Sigma Aldrich) for 1 hour at 37 °C, followed by a 5-min treatment with 5 mM EDTA and 2% FCS to stop the enzymatic reaction. Cells (1 × 10^6^) were stained with a single antibody cocktail containing CD24-PerCp-Cy5.5, CD45.2 FITC, CD64-AF647, CD89-PE, Ly6G-APC-Cy7, MHC-II-Horizon V500 and Siglec F-APC-R700 (BD Bioscience) and CD11b-BV711 CD11c-PeCy, Ly6C-BV605 and Fc block (Biolegend), and acquired in the presence of the viability dye SytoxBlue (Invitrogen). Cells were gated as described in ref. ^39^ and Suppl. Fig. 1, using FlowJo 9.8.5. The cell types were defined by the following surface markers: AMφ (Siglec F^+^, CD11c^+^, CD64^+^ and CD11b^–^), interstitial macrophages (CD11b^+^, MHC-II^+^, CD64^+^, CD11c^+^ and CD24^–^), Ly6C-positive and Ly6C-negative monocytes (CD11b^+^, MHC-II^+^, CD64^+/–^ Ly6C^+^ or LyC^–^), CD103^+^ DCs (CD11c^+^, CD24^+^, CD103^+^, MHC-II^+^ and CD11b^–^), CD11b-positive DCs (CD11b^+^, MHC-II^+^, CD11c^+^, CD64^–^ and CD24^int^), neutrophils (Ly6G^+^, CD11b^+^) and eosinophils (Siglec F^+^, CD11b^+^ and CD11c^–^). In human PB, cells were defined as neutrophils (CD15^+^, CD11b^+^ and SSC^hi^) and monocytes (CD14^+^, CD15^–^, CD11b^+^, SSC^int^ and FSC^hi^). In NHP PB, cells were defined as neutrophils (CD49d^–^, SSC^hi^) and monocytes (CD14^+^, SSC^int^).

### Quantitative mRNA analysis of the lung

The left lung lobe from day 5 of infection was weighed, and 30 mg of tissue was homogenised in 600 μl of RT lysis buffer containing 6 μl of β-mercaptoethanol and 3 μl of Reagents DX (Qiagen), using Qiagen TissueLyzer LT with 5-mm beads set to 50 Hz for 2 × 2.5 min. The beads had previously been soaked in 1 M NaOH and washed 5× in ethanol and 2× in lysis buffer to remove potential RNAse contamination. RNA was extracted with the RNAeasy mini kit (Qiagen) according to the manufacturer’s instructions, including the DNase digestion step. The total RNA was eluted two times in 30 μl of RNAse-free water. RNA concentration was measured by Nanodrop and stored at −80 °C until analysis. cDNA was generated with 500 ng of PR8 or 750 ng of Cal7 RNA, using the RT² First Strand Kit (Qiagen) in 15-μl reactions after elimination of genomic DNA according to the manufacturer’s instructions. Real-time PCR was performed using a Quant Studio7 Real Time PCR System.

For the detection of 26 mouse genes of interest and three housekeeping genes (*tbp*, *gaph* and *ubc*), the specific primers were provided in a custom-made RT2-Profiler array (Qiagen) and amplified with RT² SYBR Green ROX qPCR Mastermix (Qiagen). LinRegPCR software (Version 2014.x) was used for baseline correction, efficiency calculation and Cq calculation. q-BASE + software package (Version 3.1, Biogazelle) was utilised to calculate the calibrated normalised relative quantities (CNRQ values) of the gene of interest by normalising across plates, using two constant housekeeping genes (*tbp* and *ubc*) and relative to a reference sample across all plates. The CNRQ values are shown.

Additional methods are described in the ‘Supplementary information' section.

### Statistics

Graphpad Prism 6.0 (GraphPad Software, San Diego, CA, USA) was used for statistical analyses and graphs. Analyses of differences between sample groups were performed using the tests indicated in the text. Data shown are means ± SEM, unless otherwise stated. *P* < 0.05 was considered statistically significant.

### Study approval

The CSL/Zoetis Animal Ethics Committee and the University of Melbourne Animal Ethics Committee approved all mouse-related procedures and protocols. Human and NHP blood was provided by the Red Cross Blood Service, Australia, under the material supply deed #15-12VIC-08 and by the Monash Animal Research Platform, Australia, respectively.

## Supplementary information

Supplementary Material

## References

[1] Bonilla FA (2015). Practice parameter for the diagnosis and management of primary immunodeficiency. J. Allergy Clin. Immunol..

[2] Picard C (2015). Primary immunodeficiency diseases: an update on the classification from the International Union of Immunological Societies Expert Committee for primary immunodeficiency 2015. J. Clin. Immunol..

[3] Jolles S (2014). Subclinical infection and dosing in primary immunodeficiencies. Clin. Exp. Immunol..

[4] Kainulainen L, Vuorinen T, Rantakokko-Jalava K, Osterback R, Ruuskanen O (2010). Recurrent and persistent respiratory tract viral infections in patients with primary hypogammaglobulinemia. J. Allergy Clin. Immunol..

[5] Peltola V, Waris M, Kainulainen L, Kero J, Ruuskanen O (2013). Virus shedding after human rhinovirus infection in children, adults and patients with hypogammaglobulinaemia. Clin. Microbiol Infect..

[6] Hart TK (2001). Preclinical efficacy and safety of mepolizumab (SB-240563), a humanized monoclonal antibody to IL-5, in cynomolgus monkeys. J. Allergy Clin. Immunol..

[7] Dall’Acqua WF, Kiener PA, Wu H (2006). Properties of human IgG1s engineered for enhanced binding to the neonatal Fc receptor (FcRn). J. Biol. Chem..

[8] Ramisse F (1998). Effective prophylaxis of influenza A virus pneumonia in mice by topical passive immunotherapy with polyvalent human immunoglobulins or F(ab’)2 fragments. Clin. Exp. Immunol..

[9] Gonzalez-Quintela A (2008). Serum levels of immunoglobulins (IgG, IgA, IgM) in a general adult population and their relationship with alcohol consumption, smoking and common metabolic abnormalities. Clin. Exp. Immunol..

[10] Longet S (2013). Human plasma-derived polymeric IgA and IgM antibodies associate with secretory component to yield biologically active secretory-like antibodies. J. Biol. Chem..

[11] Longet S (2014). Reconstituted human polyclonal plasma-derived secretory-like IgM and IgA maintain the barrier function of epithelial cells infected with an enteropathogen. J. Biol. Chem..

[12] Hong DK, Tremoulet AH, Burns JC, Lewis DB (2011). Cross-reactive neutralizing antibody against pandemic 2009 H1N1 influenza a virus in intravenous immunoglobulin preparations. Pediatr. Infect. Dis. J..

[13] Rockman S (2017). Intravenous immunoglobulin protects against severe pandemic influenza infection. EBioMedicine.

[14] Jegaskanda S (2014). Cross-reactive influenza-specific antibody-dependent cellular cytotoxicity in intravenous immunoglobulin as a potential therapeutic against emerging influenza viruses. J. Infect. Dis..

[15] Monteiro RC, Van De Winkel JG (2003). IgA Fc receptors. Annu. Rev. Immunol..

[16] Wilson TJ, Fuchs A, Colonna M (2012). Cutting edge: human FcRL4 and FcRL5 are receptors for IgA and IgG. J. Immunol..

[17] Bidgood SR, Tam JC, McEwan WA, Mallery DL, James LC (2014). Translocalized IgA mediates neutralization and stimulates innate immunity inside infected cells. Proc. Natl Acad. Sci. USA.

[18] van Egmond M (1999). Human immunoglobulin A receptor (FcalphaRI, CD89) function in transgenic mice requires both FcR gamma chain and CR3 (CD11b/CD18). Blood.

[19] van Egmond M (2000). FcalphaRI-positive liver Kupffer cells: reappraisal of the function of immunoglobulin A in immunity. Nat. Med..

[20] Stewart WW, Kerr MA (1990). The specificity of the human neutrophil IgA receptor (Fc alpha R) determined by measurement of chemiluminescence induced by serum or secretory IgA1 or IgA2. Immunology.

[21] Bakema JE, van Egmond M (2011). The human immunoglobulin A Fc receptor FcalphaRI: a multifaceted regulator of mucosal immunity. Mucosal Immunol..

[22] Monteiro RC (2014). Immunoglobulin A as an anti-inflammatory agent. Clin. Exp. Immunol..

[23] Wu J (2007). Fc RI (CD89) alleles determine the proinflammatory potential of serum IgA. J. Immunol..

[24] Maillet A (2011). The airways, a novel route for delivering monoclonal antibodies to treat lung tumors. Pharm. Res.

[25] Bioley G (2017). Plasma-derived polyreactive secretory-like IgA and IgM opsonizing *Salmonella enterica* typhimurium reduces invasion and gut tissue inflammation through agglutination. Front. Immunol..

[26] Launay P (2000). Fcalpha receptor (CD89) mediates the development of immunoglobulin A (IgA) nephropathy (Berger’s disease). Evidence for pathogenic soluble receptor-Iga complexes in patients and CD89 transgenic mice. J. Exp. Med..

[27] Xu L (2016). Critical role of Kupffer cell CD89 expression in experimental IgA nephropathy. PloS One.

[28] Pasquier B (2005). Identification of FcalphaRI as an inhibitory receptor that controls inflammation: dual role of FcRgamma ITAM. Immunity.

[29] Jackson RJ (2013). The current status of vertebrate cellular mRNA IRESs. Cold Spring Harb. Perspect. Biol..

[30] Pommerenke C (2012). Global transcriptome analysis in influenza-infected mouse lungs reveals the kinetics of innate and adaptive host immune responses. PloS One.

[31] Kanamaru Y (2008). Inhibitory ITAM signaling by Fc RI-FcR chain controls multiple activating responses and prevents renal inflammation. J. Immunol..

[32] Kelton W (2014). IgGA: a “cross-isotype” engineered human Fc antibody domain that displays both IgG-like and IgA-like effector functions. Chem. Biol..

[33] He W (2017). Alveolar macrophages are critical for broadly-reactive antibody-mediated protection against influenza A virus in mice. Nat. Commun..

[34] Shaykhiev R (2009). Smoking-dependent reprogramming of alveolar macrophage polarization: implication for pathogenesis of chronic obstructive pulmonary disease. J. Immunol..

[35] Lindh E (1975). Increased risistance of immunoglobulin A dimers to proteolytic degradation after binding of secretory component. J. Immunol..

[36] Renegar KB, Jackson GD, Mestecky J (1998). In vitro comparison of the biologic activities of monoclonal monomeric IgA, polymeric IgA, and secretory IgA. J. Immunol..

[37] Ye J (2010). Intranasal delivery of an IgA monoclonal antibody effective against sublethal H5N1 influenza virus infection in mice. Clin. Vaccin Immunol..

[38] Rossato E (2015). Reversal of arthritis by human monomeric IgA through the receptor-mediated SH2 domain-containing phosphatase 1 inhibitory pathway. Arthritis Rheum..

[39] Misharin AV, Morales-Nebreda L, Mutlu GM, Budinger GR, Perlman H (2013). Flow cytometric analysis of macrophages and dendritic cell subsets in the mouse lung. Am. J. Respir. Cell Mol. Biol..

