# Supplementary material for: Topical application of human-derived Ig isotypes for the control of acute respiratory infection evaluated in a human CD89-expressing mouse model
Source: Mucosal Immunol. 2019 May 19;12(4):1013–24. doi: 10.1038/s41385-019-0167-z (PMC7746524; doi:10.1038/s41385-019-0167-z)
Supplement: Supplementary file 1 — Supplementary Material [file 41385_2019_167_MOESM1_ESM.doc]

**Supplementary Material**

**Supplementary Table I. Human plasma-derived immunoglobulin preparations.**

| **Constituent^a^** | **IgG** | **mIgA** | **IgAM** | **SIgAM** |
| --- | --- | --- | --- | --- |
| **IgA** | 0.004 | 98.0 | 65.2 | 59.3 |
| **IgM** | 1.5 | 1.7 | 34.8 | 31.6 |
| **IgG** | >98 | 0.3 | <0.02 | <0.02 |
| **Monomers** | 99.8^b^ | 61.2 | 23.1 | 23 |
| **Dimers** | - | 18.2 | 16.7 | 17 |
| **Polymers** | <0.1 | 16.6 | 55.6 | 55 |
| **IgA with J-chain** | 0 | 9 | 47 | 47 |
| **Ig with J-chain** | 0 | 11 | 65 | 65 |
| **recSC** | 0 | 0 | 0 | 9.1 |

^a^ Constituents are expressed in percentage.

^b^ Includes dimers.

**Supplementary Figures:**

**Supplementary Figure 1. CD89 sequence.** The CD89 sequence in the CD89^tg/wt^ line contains three nucleotide changes: 161AàT, 337 GàA and 538 TàC, resulting in two amino acid changes: glutamic acid to valine, 54EàV and aspartic acid to asparagine, 114DàN. The signal peptide is encoded by both S1 and S2 sequences. Ig-like domains (EC1, EC2) transmembrane (TM) and cytosolic tail (C) are indicated.

**Supplementary Figure 2. mCherry and lack of GFP expression in CD89^tg/wt^ and CD89^tg/wt^ / LyzM^cre/wt^ mice.** Peripheral blood myeloid cells (a) and lymphoid cells (b) from CD89^tg/twt^parent (open grey), CD89^tg/wt^ / LyzM^cre/wt^ progeny (black) and littermates (filled grey) were tested for mCherry and GFP expression levels. mCherry expression was downregulated in neutrophils and monocytes in CD89^tg/wt^ / LyzM^cre/wt^ progeny (black). GFP was undetectable in all tested cell types.

**Supplementary Figure 3.** Flow cytometry gating strategy of single cell suspension from whole lung of CD89^tg/wt^ / LyzM^cre/wt^ mice to characterise CD89 expression in Figure 5.

**Supplementary Figure 4.** CD89 expression levels in human PB eosinophils, neutrophils and CD14 positive monocytes from four donors. Fluorescence minus one (FMO) controls are shown in grey.

**Supplementary Figure 5.** recSC associates with IgA and IgM in the SIgAM preparation.

Recombinant secretory component (recSC, 79.6 kDa) was mixed with IgAM at weight ratio of 1:10 and 1:3 and treated as indicated in (a) and the Supplementary Methods section. An equal amount of recSC, as in the SIgAM (1:3)­­ preparation, was run as control.

(b) SIgAM samples and recSC protein, final flow-through and eluates from the two columns were separated on SDS-PAGE under denaturing conditions. After protein transfer onto nitrocellulose, the membrane was probed with anti-human SC antibody.

In SIgAM (1:10), recSC was bound to IgA and IgM with very little excess recSC present in the flow-through (lane 4), as recSC was present in the eluates of the IgA (lane 7) and IgM (lane 10) columns, respectively. In comparison, in SIgAM (1:3) increased excess of recSC was present in flow-through (lane 5), indicating that a weight ratio of recSC:IgAM of 1:10 facilitated optimal association of recSC to the J-chain containing polymeric IgA and IgM in IgAM.

**Supplementary Methods**

***Whole genome sequencing (WGS)***

Genomic DNA was extracted from a spleen sample of line 5 using DNAeasy kit (Qiagen, Cat. No 69504) and then processed using TruSeq chemistry (Illumina, San Diego, CA). Paired-end sequencing was performed using HiSeq Illumina platform and the CASAVA pipeline v1.8.2 produced a library of 245,330,061 PE reads of 100bp. A hybrid Tg reference genome was created using the GRCm38.p3 mouse reference genome from NCBI (NCBI Resource Coordinators, 2017) and the sequence of the knock-in vector. Bowtie2 v2.2.4^1^ was used to map reads, the Integrative Genomics Viewer^2^ to visualize mapped reads, Socrates v0.99.0^3^ to call structural variants, Control-FREEC v7.2^4^ to call copy-number variations and the *mpileup* function in samtools v1.2^5^ to call SNPs. A custom script was written to validate and annotate integration events and fusion points. The genomic sequence is accessible under BioProject ID PRJNA531719 in the NCBI's SRA repository.

***Preparation of human plasma-derived immunoglobulins***

Human plasma-derived Ig formulations were prepared as previously reported^7^. mIgA (primarily monomeric IgA) and IgAM (a mixture of primarily dimeric/polymeric IgA and IgM) were purified from process intermediates of IgG immunoglobulins manufactured from human plasma, by a sequence of retaining ion exchange and depleting affinity chromatography. All Ig preparations originated from a single plasma pool collected between 2011-2013. The IgG products were composed of 97% monomeric IgG and concentrated to either 100 mg/ml (IgPro10, Privigen®) or 200 mg/ml (IgPro20, Hizentra®). The mIgA preparation was 98% IgA, from which >90% was monomeric or non-covalently polymeric (no J-chain link) IgA. The IgA/M (IgAM) solution contained pIgA and IgM in a 2:1 mass ratio (Suppl. Table I). In IgAM, presumably all of IgM, and about 47% of IgA contained a covalent J-chain link (Suppl. Table I).

The recombinant secretory component (recSC) fragment was expressed in CHO cells and purified via diafiltration to 1 mg/ml. The recSC (79.6 k­­Da) was then mixed into the IgAM fraction at a mass ratio of 1:10 for 50 min at room temperature in PBS in order to fully associate with the J-chain-containing polymeric IgA and IgM to produce SIgAM. All IgA-containing preparations (mIgA, IgAM, SIgAM) were formulated at 50 mg/ml in 125 mM proline. All structural characteristics provided in Supplementary Table 1, were stable from batch to batch preparation and over tested time period.

***Analysis of recSC association with IgAM***

The final SIgAM preparation, as prepared above, and a test SIgAM preparation obtained with a mass ratio of recSC to IgAM of 1:3, and recSC alone, were run over two consecutive affinity chromatography columns, CaptureSelect™ IgA and CaptureSelect™ IgM (Thermofisher), respectively. Starting protein samples, flow-through and eluates of columns were separated under denaturing conditions on a polyacrylamide gel and transferred onto nitrocellulose membrane. recSC was detected with a HRP-conjugate of goat anti-human SC (ABIN458053, antibodies-online.com) as shown in Supplementary Figure 5.

***Generation of BMDMs***

BMDM were generated from CD89^tg/wt^ / LyzM^cre/wt^ and littermate controls. Mouse tibiae and femurs were flushed with cold PBS through a 70 μm-wide cut off cell strainer and centrifuged. Erythrocytes were lysed with ammonium chloride and residual cells were resuspended in 10 ml of complete RPMI (c-RPMI, Sigma-Aldrich), 10% FCS (Sigma-Aldrich), 100 U/ml penicillin/streptomycin (Life technologies), 1x Glutamax (Life technologies) and incubated overnight in a 10 cm non-tissue culture treated dish (Corning). The next day, non-adherent cells were seeded at a density of 2 x 10^6^ in c-RPMI supplemented with 40 ng/ml M-CSF (Prospec) into either 8 well glass slide chambers (Lab-Tek II CC2™) for phagocytosis assays or into 10 cm tissue culture-treated dishes (Corning) for stimulation, phosphorylation and immunoprecipitation assays. Fresh M-CSF was added every 2-3 days. BMDM were harvested with a scraper after a 20 min incubation with Accutase (Sigma-Aldrich) on day 7-8 for experimentation. Typically, 70-90 % of CD89^tg/wt^ / LyzM^cre/wt^ BMDM expressed CD89.

***Cells and cell lines***

MDCK cells were maintained in RPMI 1640 without glutamine (Sigma-Aldrich) supplemented with 10% (vol/vol) heat-inactivated FCS (HyClone), benzylpenicillin (100 IU/ml; CSL Ltd.), streptomycin sulfate (180 g/ml; MP Biomedicals), gentamicin sulfate (24 g/ml; Pfizer), glutamine (2 mM; Sigma-Aldrich), and sodium pyruvate (2 mM; MP Biomedicals). Similar medium without FCS, L-glutamine, and sodium pyruvate was used for dilutions, and RPMI containing 1 mg/ml of bovine serum albumin (BSA) was used as a control for virus neutralization assays. Human white blood cells were isolated from healthy volunteers and non-human primate peripheral blood cells were generated by 7 min RBC lysis with ammonium chloride solution.

***Viruses***

A/Puerto Rico/8/34 (PR8; H1N1) and A/California/7/09 (Cal7; H1N1) influenza viruses were propagated in 10-day-old embryonated hen’s eggs at 35°C for 2 days. Allantoic fluid was then harvested and stored at -80°C.

***Hemagglutination inhibition (HI) assay***

HI assays were performed by standard procedures^8^ in round-bottom polystyrene microtitre plates (Nunc, Denmark) using 1% chicken red blood cells. Titres are expressed as the reciprocal of the highest dilution of sample that inhibited 4 hemagglutinating units (HAU) of virus.

***Virus plaque-forming assay and micro neutralisation assay***

Plaque formation in MDCK cell monolayers^9^ cultured in 6-well tissue culture plates was used to measure viral titres in lung extracts and neutralization of virus by Ig preparations. For neutralization assays, viruses were prepared in RPMI medium and then mixed with decreasing concentrations of Ig preparations at a ratio of 1:9 (vol/vol). The mixtures were then incubated at 37°C for 30 min before being added to confluent monolayers of MDCK cells and allowed to adsorb for 45 min at 37°C. The cells were overlaid with 9 mg/ml agarose (Sigma-Aldrich) with 2 μg/ml L-(tosylamido-2-phenyl) ethyl chloromethyl ketone (TPCK)-treated trypsin (Worthington Biochemical) in Leibovitz L-15 medium with glutamine (Gibco) at pH6.8, containing 0.4 mM HEPES buffer, 0.028% NaHCO_3_, 120 μg/ml penicillin, and 200 μg/ml streptomycin. After 3 days of incubation at 37°C in 5% CO_2_, plaques were counted without staining. The virus neutralization by Ig preparations was calculated as the percentage reduction in the number of plaques present in the presence of Ig compared to the PBS controls.

***Neuraminidase Inhibition assay***

An enzyme-linked lectin assay was used to determine NA activity and inhibition by antibody^10^. Briefly, 96-well Maxisorp plate (Thermo Fisher, Denmark) was coated with 100 µl of a 25 µg/ml solution of bovine fetuin (Sigma Aldrich, USA) in PBS and left overnight at 4^o^ C. A 0.5% stock of each antibody preparation (5 mg/ml) was serially diluted two-fold in PBS then incubated with an equal volume of either PR8 or Cal7 virus (at a previously determined concentration yielding 95% of maximal sialidase activity) at room temperature for 45 min alongside PBS and virus alone controls. The fetuin-coated plate was washed once with PBS and then 100 µl/well of each dilution was added in duplicate before the plate was incubated at 37º C for 16 h. The plate was then washed four times in PBS with 0.05% Tween-20 (PBST) then incubated for one hour at room temperature with peanut *Arachis Hypogaea* lectin conjugated to HRP (Cosmo Bio, Japan) to detect galactose residues revealed by the removal of sialic acid. An additional five washes in PBST were performed before 100 µl/well of SureBlue tetramethylbenzidine substrate (Seracare, United States) was added and left at room temperature for 20 min. The reaction was stopped with 50 µl/well of 1M HCl and read at 450 nm on a Thermo Fisher Multiskan Ascent plate reader.

***Detection of IgA in BAL***

IgA in BAL was detected by ELISA as follows. Coating, blocking and detection steps were 1 h each (at room temperature). Sample incubation was 2 h at room temperature. Goat anti-human IgA Ab (A80-202A; Bethyl) was coated onto a microplate (NUNC, Maxisorp) at 0.5 μg/ml final concentration in PBS supplemented with 1% BSA (which also served as blocking buffer). Goat anti-human IgA detection Ab (A80-202P; Bethyl) conjugated with HRP was used at a 0.3 μg/ml final concentration (low cross buffer (Candor), 0.1% casein buffer). Tetramethylbenzidine was added to the wells for 10 min at room temperature. After stopping the reaction, signal was measured at OD = 450 nm using an ELISA plate reader (Perkin Elmer, Envision Xcite).

**References**

1. Langmead B, Salzberg SL. Fast gapped-read alignment with Bowtie 2. *Nat Methods* 2012; **9**(4)**:** 357-359.

2. Robinson JT, Thorvaldsdottir H, Winckler W, Guttman M, Lander ES, Getz G *et al.* Integrative genomics viewer. *Nat Biotechnol* 2011; **29**(1)**:** 24-26.

3. Schroder J, Hsu A, Boyle SE, Macintyre G, Cmero M, Tothill RW *et al.* Socrates: identification of genomic rearrangements in tumour genomes by re-aligning soft clipped reads. *Bioinformatics* 2014; **30**(8)**:** 1064-1072.

4. Boeva V, Popova T, Bleakley K, Chiche P, Cappo J, Schleiermacher G *et al.* Control-FREEC: a tool for assessing copy number and allelic content using next-generation sequencing data. *Bioinformatics* 2012; **28**(3)**:** 423-425.

5. Li H, Handsaker B, Wysoker A, Fennell T, Ruan J, Homer N *et al.* The Sequence Alignment/Map format and SAMtools. *Bioinformatics* 2009; **25**(16)**:** 2078-2079.

6. Longet S, Vonarburg C, Lotscher M, Miescher S, Zuercher A, Corthesy B. Reconstituted human polyclonal plasma-derived secretory-like IgM and IgA maintain the barrier function of epithelial cells infected with an enteropathogen. *J Biol Chem* 2014; **289**(31)**:** 21617-21626.

7. Bioley G, Monnerat J, Lotscher M, Vonarburg C, Zuercher A, Corthesy B. Plasma-Derived Polyreactive Secretory-Like IgA and IgM Opsonizing Salmonella enterica Typhimurium Reduces Invasion and Gut Tissue Inflammation through Agglutination. *Front Immunol* 2017; **8:** 1043.

8. Dowdle WR, Kendal AP, Noble GR. Influenza viruses. In: Lennette EH, Schmidt NJ (eds). *Diagnostic procedures for viral, rickettsial, and chlamydial infections.* . American Public Health Association: Washington, DC, 1979, pp 585–609.

9. Tannock GA, Paul JA, Barry RD. Relative immunogenicity of the cold-adapted influenza virus A/Ann Arbor/6/60 (A/AA/6/60-ca), recombinants of A/AA/6/60-ca, and parental strains with similar surface antigens. *Infect Immun* 1984; **43**(2)**:** 457-462.

10. Eichelberger MC, Couzens L, Gao Y, Levine M, Katz J, Wagner R *et al.* Comparability of neuraminidase inhibition antibody titers measured by enzyme-linked lectin assay (ELLA) for the analysis of influenza vaccine immunogenicity. *Vaccine* 2016; **34**(4)**:** 458-465.
